# Vacant Appointments: Canvassing or Non-Canvassing for Elections

**Published:** 1914-12-12

**Authors:** 


*To the Editor of* THE HOSPITAL.

### Vacant Appointments: Canvassing or Non-Canvassing for Elections.

*To the Editor of* THE HOSPITAL.

SIR,—Your comments at the foot of my letter appearing in THE HOSPITAL this week prompt me to inflict this further communication upon you.

I do not feel that I have missed the point of your comments. The contention that where a committee forbids canvassing it should enforce its decision, no matter how foolish that decision may be, is an obvious fact, but I am tilting against the absurdity of the insertion of such a prohibition which you seem to favour, or at any rate to discuss with equanimity. Indeed, you even go so far as to recommend that a clause forbidding canvassing should be inserted in every advertisement. If there is no such prohibition there is no temptation to evade it. What would the country say if members of Parliament were to present themselves for election but were not permitted to lay their merits before the electorate, and the electors not permitted to see the candidate?

I regret you seem in doubt as to my meaning. It is that the candidate shall be free to canvass and the electors free to interview and inquire into the merits of the candidates.—Yours truly,

December 4, 1914. A HOSPITAL SECRETARY.

[*** We are glad our correspondent now states plainly he is opposed to a prohibition against canvassing being inserted in the advertisements for candidates for hospital appointments. On this side of the question many good points may be urged. On the other hand, sound arguments have been formulated in favour of the abolition of all canvassing for members of Parliament having regard to the abuses which at present attach to canvassing and the selection as candidates of aliens, nominees of trades and syndicates, and other types of unworthiness, who too frequently procure seats in the House of Commons under the existing system. We are wholly in favour of entire freedom in these matters, but, to secure it, an open system of election must itself be freed from the abuses which at present disgrace and even degrade the political electorate. Whatever system is followed our voluntary hospitals must be absolutely protected from similar abuses. Does our correspondent realise that up to forty years ago, before reforms were introduced, an election to the post of honorary medical officer to the larger voluntary hospitals, especially in the provinces, was as costly, full of abuses and conflicting influences, as a Parliamentary election?—Ed. The Hospital.]

